# Supplementary material for: Chloroplast and whole-genome sequencing shed light on the evolutionary history and phenotypic diversification of peanuts
Source: Nat Genet. 2024 Aug 13;56(9):1975–84. doi: 10.1038/s41588-024-01876-7 (PMC11387195; doi:10.1038/s41588-024-01876-7)
Supplement: Supplementary file 1 — Supplementary Figs. 1–15. [file 41588_2024_1876_MOESM1_ESM.pdf]

nature genetics

Supplementary information https://doi.org/10.1038/s41588-024-01876-7

Chloroplast and whole-genome sequencing shed light on the evolutionary history and phenotypic diversification of peanuts

In the format provided by the authors and unedited

Supplementary Figure Legends

Supplementary Figure 1. Validation of chloroplast DNA polymorphisms. In total, five polymorphisms were validated with Sanger sequencing (a-d) and/or KASP technology (e-h).

Supplementary Figure 2. Pedigree information of the accession N524. The red and blue lines indicate the female and male parent, respectively. The accession N524 inherited the chloroplast genome from N744.

Supplementary Figure 3. Chloroplast phylogenetic tree based on mononucleotide repeat (MNR) loci.

Supplementary Figure 4. Genome-specific nuclear phylogenetic trees. (a) phylogenetic tree for 11 *A. duranensis* accessions and chromosomes 1-10 (A genome) of tetraploid peanut accessions; (b) polygenetic tree for the *A. ipaensis* accession PI468322 and chromosomes 11-20 (B genome) of tetraploid peanut.

Supplementary Figure 5. The distribution of SNPs between groups (P_B) and shared across groups (P_A). Data are presented for chromosomes 1-10 (a, c) and chromosomes 11-20 (b, d).

Supplementary Figure 6. Nucleotide diversity (Pi) for different types of peanut. The A and B sub-genomes were analyzed separately. SubA refers to the A genome (a) and SubB to the B genome (b). The accession number for each type: *A.monticola* 2, var. *peruviana* 2, var. *hypogaea* 85, var. *hirsuta* 12, var. *fastigiata* 26, var. *vulgaris* 84, var. *hypogaea* irregular 100, var. *fastigiata* irregular 44. The nucleotide diversity (Pi) was calculated using a 200 kb window with a step size of 100 kb. In the box plots, centerline indicates the median; box lower and upper edges indicate the 25% and 75% quartiles, respectively; whiskers indicate 1.5* IQR; points indicate outliers.

Supplementary Figure 7. Haplotype blocks for different peanut botanical types. Graphical block structure representation of 20 chromosomes. Haplotypes were computed with adaptive mode using window sizes of 5, 10, 20 and 50 markers and target coverage of 90%.

Supplementary Figure 8. Fine mapping of a QTL for the flowering pattern (*qFTA12*). KASP markers used for fine mapping are named according to their chromosomal position on chr.12. Phenotypes and genotypes are reported for the parental lines and 13 recombinant RILs.

Supplementary Figure 9. Features of the mutations (Mu) identified for the gene *AhTFL1*. a) Sequence alignment showing the 214 bp MITE insertion of the mutation type 1 (Mu 1); b) Integrative Genomics Viewer (IGV) image of the genomic region showing paired-end reads mapped on the candidate gene with a 1492 bp deletion described as mutation type 2 (Mu 2); c) paired-end reads mapped on the candidate gene with a 1 bp deletion (with a C missing) described as mutation type 3 (Mu 3).

Supplementary Figure 10. Fine mapping of a QTL for the inner integument color (*qIICA05*). KASP markers used for fine mapping are named according to their chromosomal position on chr05. (a) Fine mapping based on from the YZ9102×wt09-0023 RIL population. Phenotypes and genotypes are reported for the parental lines and 27 recombinant RILs. (b) Fine mapping based on the P573×P602 F_2 population. Phenotypes and genotypes are reported for the parental lines and 34 recombinant individuals.

Supplementary Figure 11. Features of the mutations (Mu) identified for the gene *AhLAC.* a) Sequence alignment showing the 214 bp insertion of the mutation type 1 (Mu 1); b) Integrative Genomics Viewer (IGV) image of the genomic region showing paired-end reads mapped on the candidate gene with a 1 bp insertion (purple I) described as mutation type 2 (Mu 2).

Supplementary Figure 12. *AhLAC* functional characterization. a-c) Seed phenotype (a), lightness (L*) level (b) and *AhLAC* expression level (c) in the *Arabidopsis* wild type line Col-0, the *Atlac*/*Att10* mutant line and four independent *35S::AhLAC* transgenic lines in the mutant background; (d-e) Tegument color (d) and epicatechin content (e) of the peanut accessions YH154, YH37, YH76 and ZYH109. In b), c) and e), data are given as mean ± SEM and the two tailed Student's *t* test was used to compare means. In the bar charts b) and e), n=3 biologically independent samples; In the bar chart c), n=6 biologically independent samples for *35S::AhLAC* transgenic lines and n=3 biologically independent samples for the *Arabidopsis Atlac/Att10* mutant and the wildtype.

Supplementary Figure 13. **Fine mapping of a QTL for the growth habit (*qGhA15*).** KASP markers used for fine mapping are named according to their chromosomal position on chr15. Phenotypes and genotypes are reported for the parental lines and 11 recombinant RILs.

Supplementary Figure 14. Features of the mutations (Mu) identified for the gene *AhMADS-box transcription factor 6*. a) Integrative Genomics Viewer (IGV) image of the genomic region showing paired-end reads mapped on the candidate gene with a 2 bp insertion (purple 2) described as mutation type 1 (Mu 1); b) Integrative Genomics Viewer (IGV) image of the genomic region showing paired-end reads mapped on the candidate gene with a 1870 bp deletion described as mutation type 2 (Mu 2); c) Integrative Genomics Viewer (IGV) image of the genomic region showing paired-end reads mapped on the candidate gene with a MITE insertion described as mutation type 3 (Mu 3).

Supplementary Figure 15. GWAS for seed oil content. Manhattan plot (left) and quantile-quantile (Q-Q) plot (right). The mixed linear model (MLM) implemented in the R package GAPIT was used to obtain statistics for each SNP locus. The horizontal line in the Manhattan plot indicates the significance $-\log_{10}(P)$ threshold for association after the Bonferroni correction. The shaded area in the Q-Q plot indicates the 95% confidence interval under the null hypothesis of no association between the SNPs and the trait, under the assumption of a uniform [0, 1] distribution for the P values.

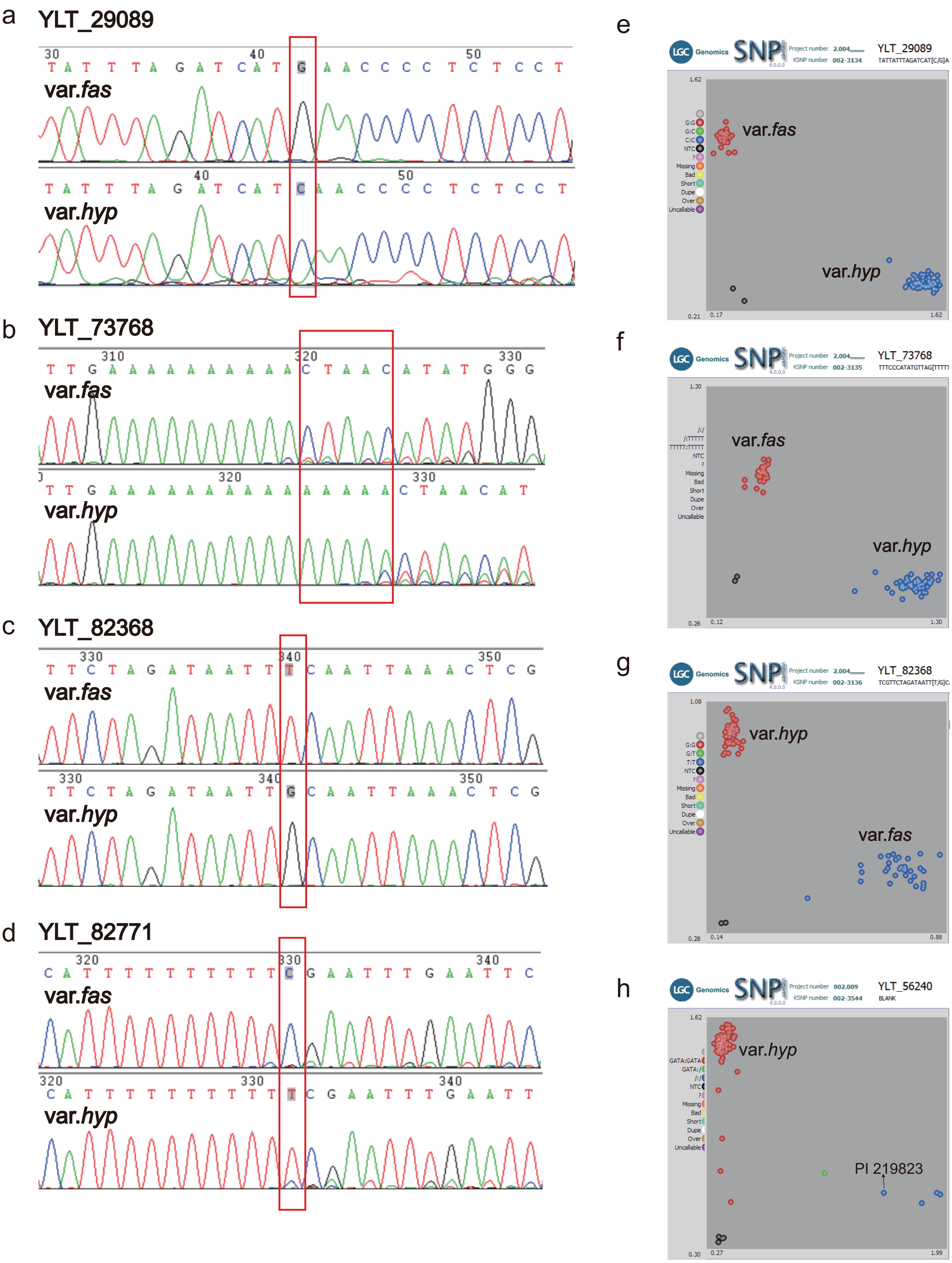

Supplementary Figure 1. Validation of chloroplast DNA polymorphisms.
In total, five polymorphisms were validated with Sanger sequencing (a-d) and/or KASP technology (e-h).

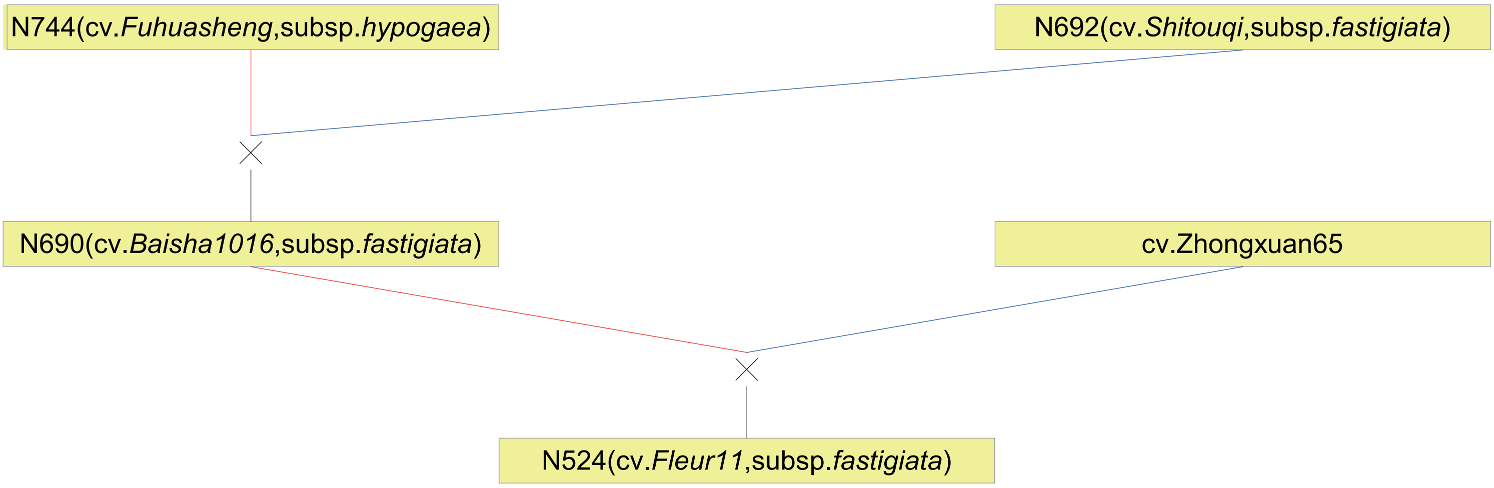

Supplementary Figure 2. Pedigree information of the accession N524. The red and blue lines indicate the female and male parent, respectively. The accession N524 inherited the chloroplast genome from N744.

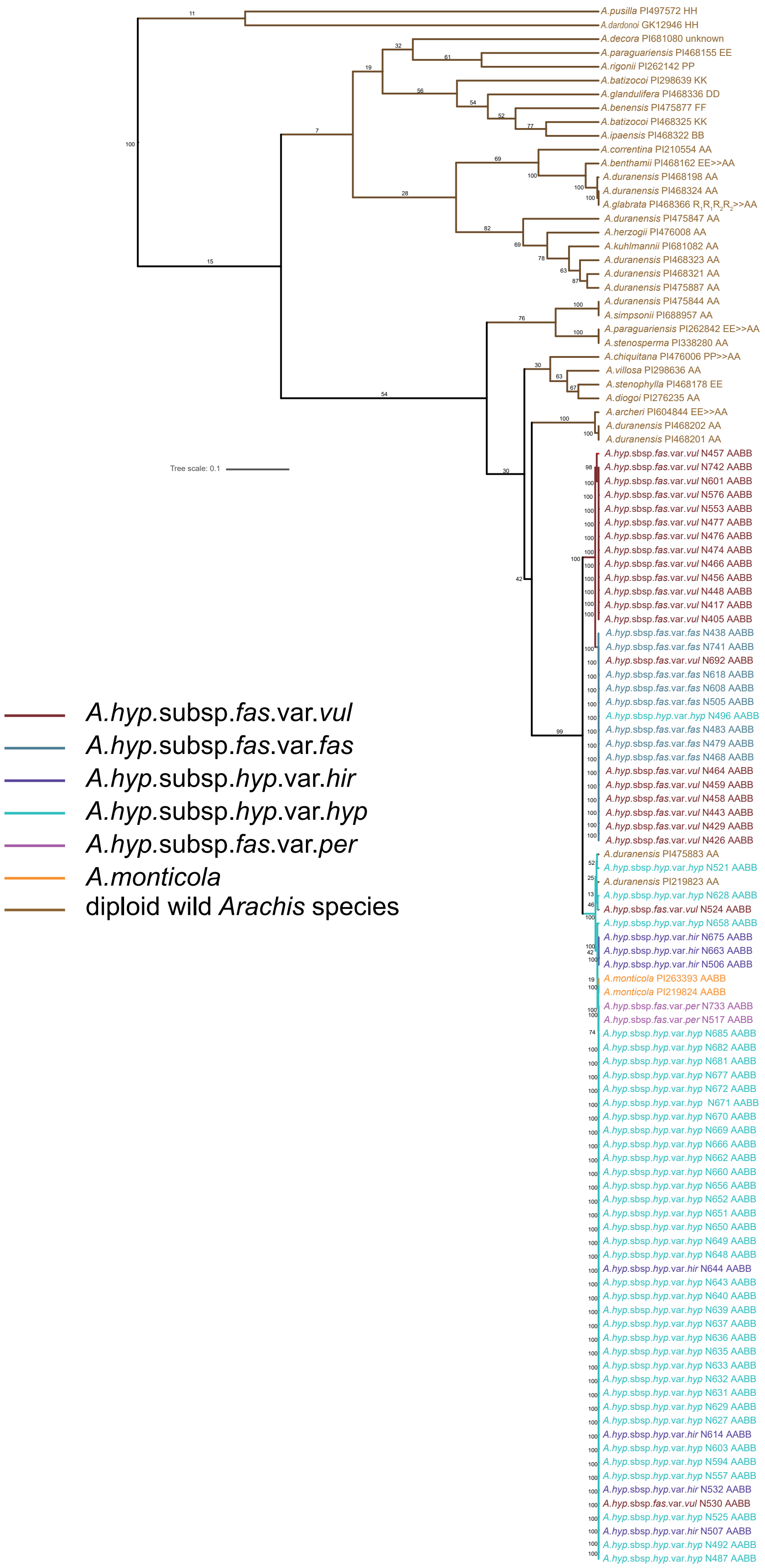

Supplementary Figure 3. Chloroplast phylogenetic tree based on mononucleotide repeat (MNR) loci.

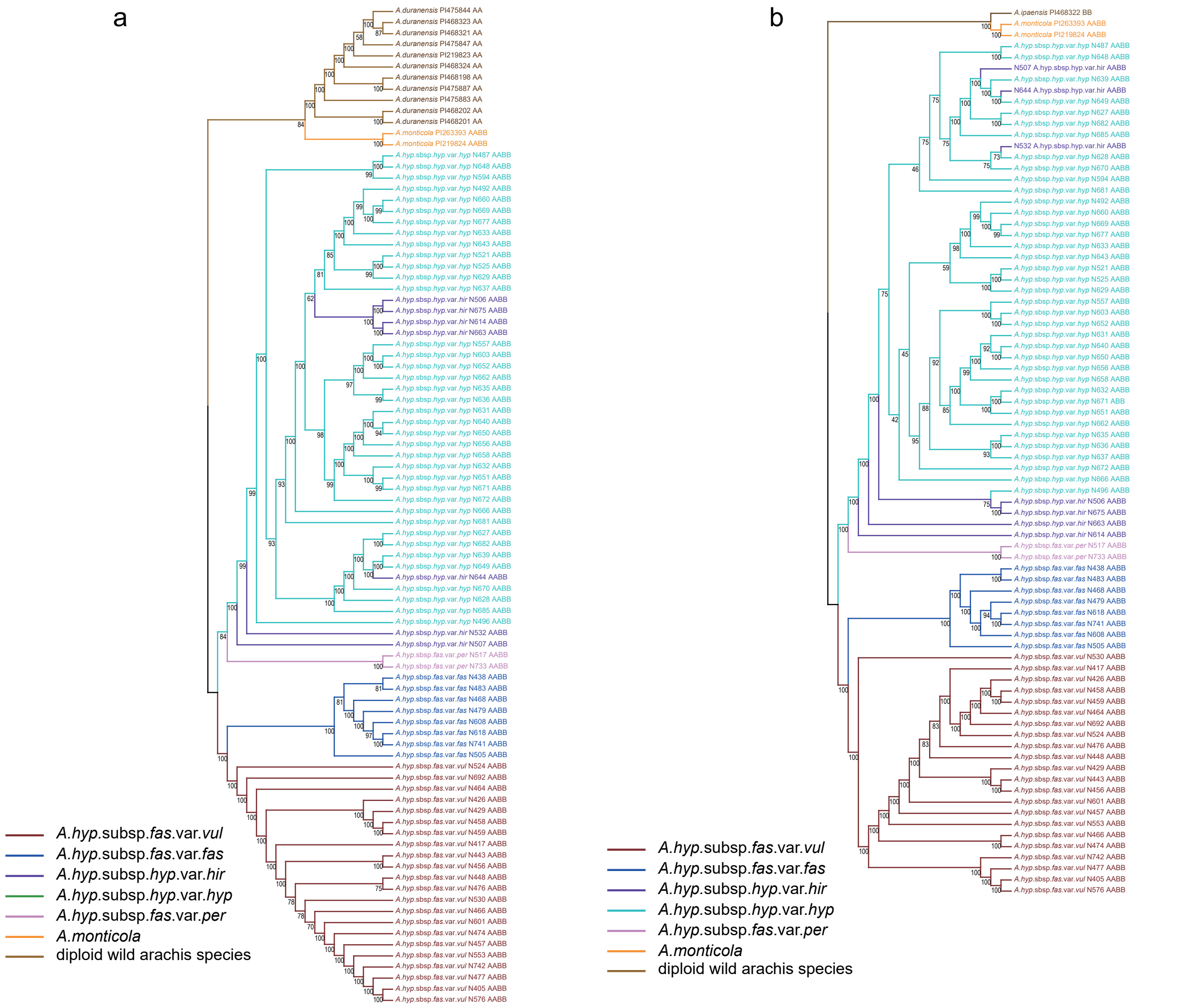

Supplementary Figure 4. Genome-specific nuclear phylogenetic trees. (a) phylogenetic tree for 11 *A. duranensis* accessions and chromosomes 1-10 (A genome) of tetraploid peanut accessions; (b) polygenetic tree for the *A. ipaensis* accession PI468322 and chromosomes 11-20 (B genome) of tetraploid peanut.

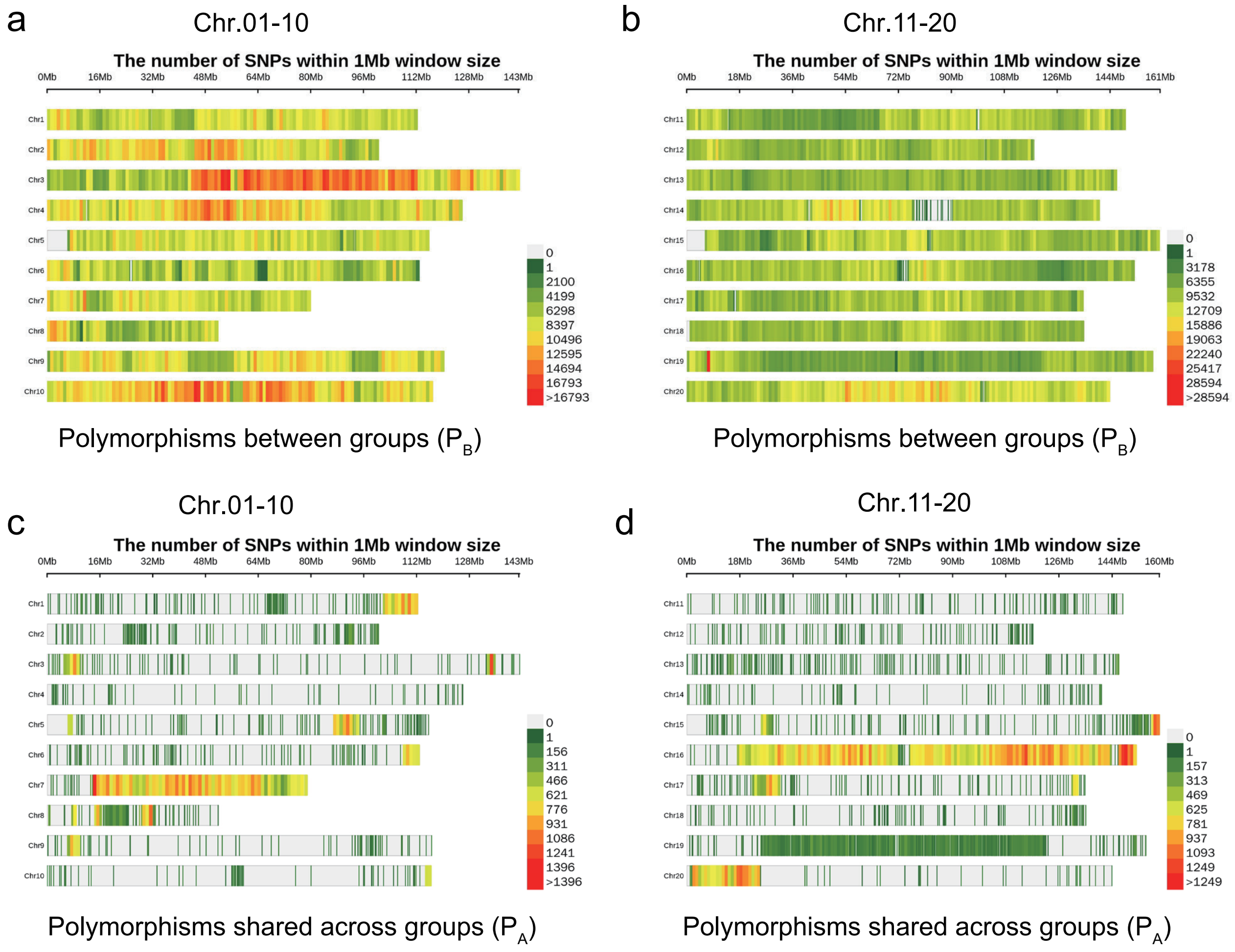

Supplementary Figure 5. The distribution of SNPs between groups (P_B) and shared across groups (P_A). Data are presented for chromosomes 1-10 (a, c) and chromosomes 11-20 (b, d).

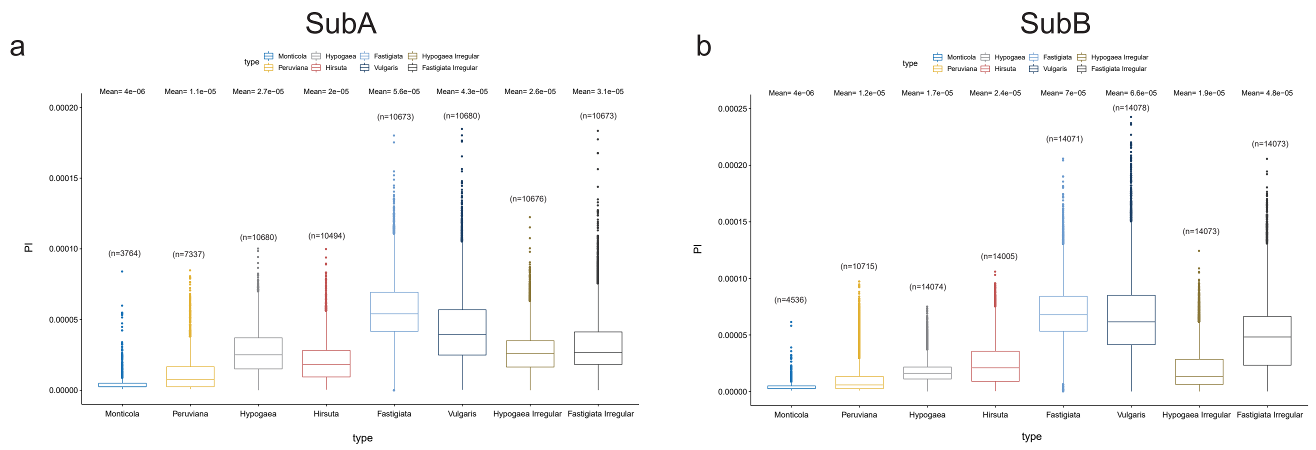

Supplementary Figure 6. Nucleotide diversity (Pi) for different peanut types. The A and B sub-genomes were analyzed separately. SubA refers to the A genome (a) and SubB to the B genome (b). The accession number for each type: *A.monticola* 2, var. *peruviana* 2, var. *hypogaea* 85, var. *hirsuta* 12, var. *fastigiata* 26, var. *vulgaris* 84, var. *hypogaea* irregular 100, var. *fastigiata* irregular 44. The nucleotide diversity (Pi) was calculated using a 200 kb window with a step size of 100 kb. In the box plots, centerline indicates the median; box lower and upper edges indicate the 25% and 75% quartiles, respectively; whiskers indicate 1.5* IQR; points indicate outliers.

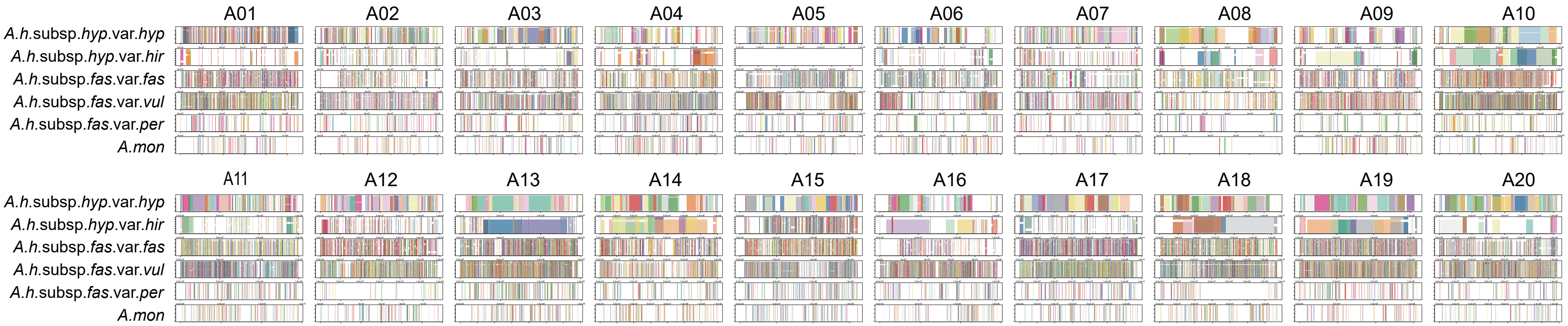

Supplementary Figure 7. Haplotype blocks for different peanut botanical types. Graphical block structure representation of 20 chromosomes. Haplotypes were computed with adaptive mode using window sizes of 5, 10, 20 and 50 markers and target coverage of 90%.

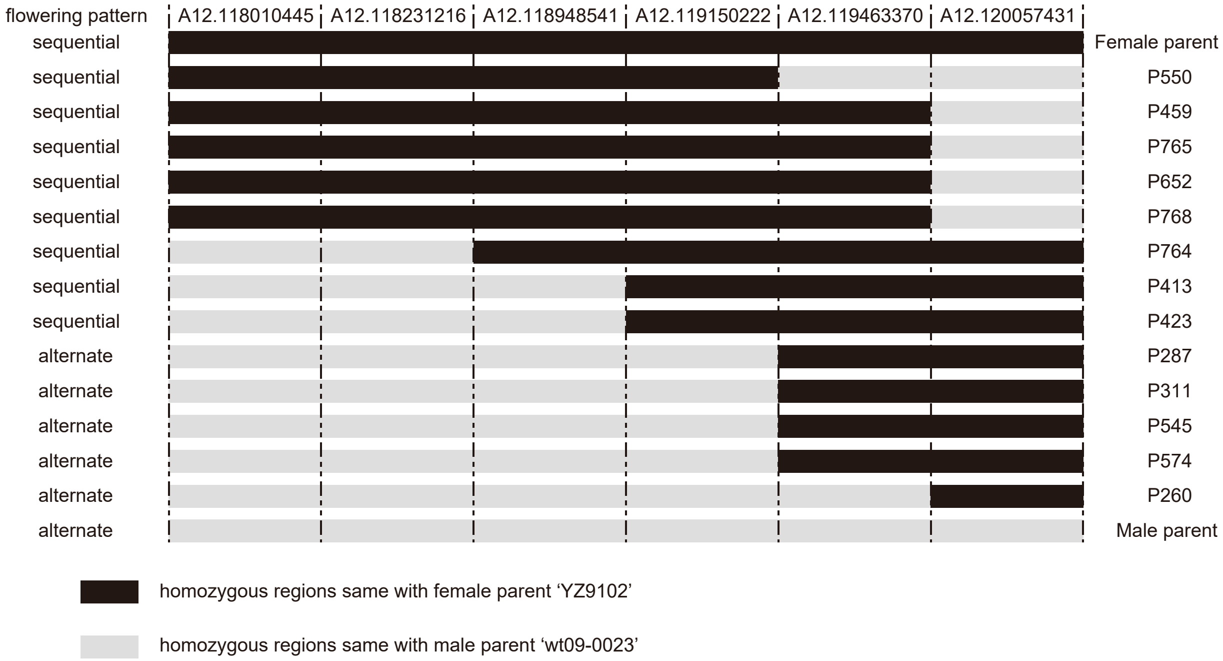

Supplementary Figure 8. Fine mapping of a QTL for the flowering pattern (*qFTA12)*. KASP markers used for fine mapping are named according to their chromosomal position on chr12. Phenotypes and genotypes are reported for the parental lines and 13 recombinant RILs.

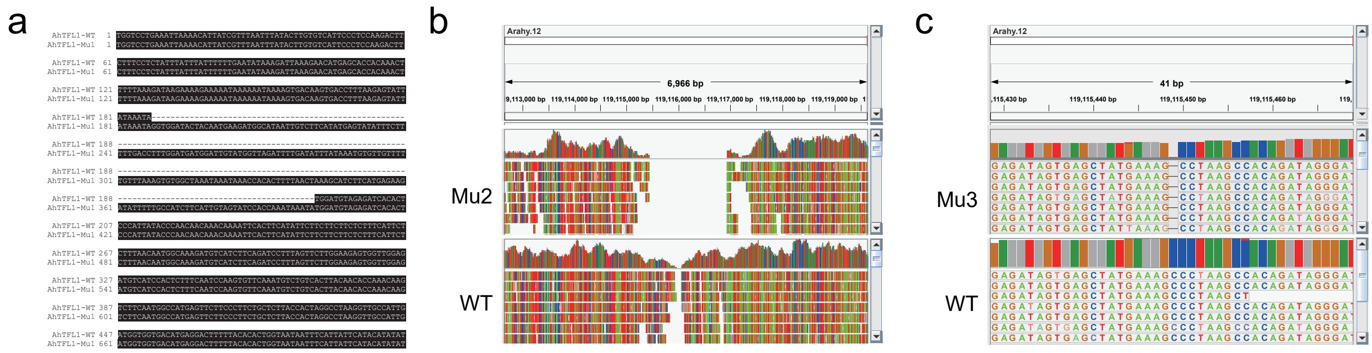

Supplementary Figure 9. Features of the mutations (Mu) identified for the gene *AhTFL1*. a) Sequence alignment showing the 214 bp MITE insertion of the mutation type 1 (Mu 1); b) Integrative Genomics Viewer (IGV) image of the genomic region showing paired-end reads mapped on the candidate gene with a 1492 bp deletion described as mutation type 2 (Mu 2); c) paired-end reads mapped on the candidate gene with a 1 bp deletion (with a C missing) described as mutation type 3 (Mu 3).

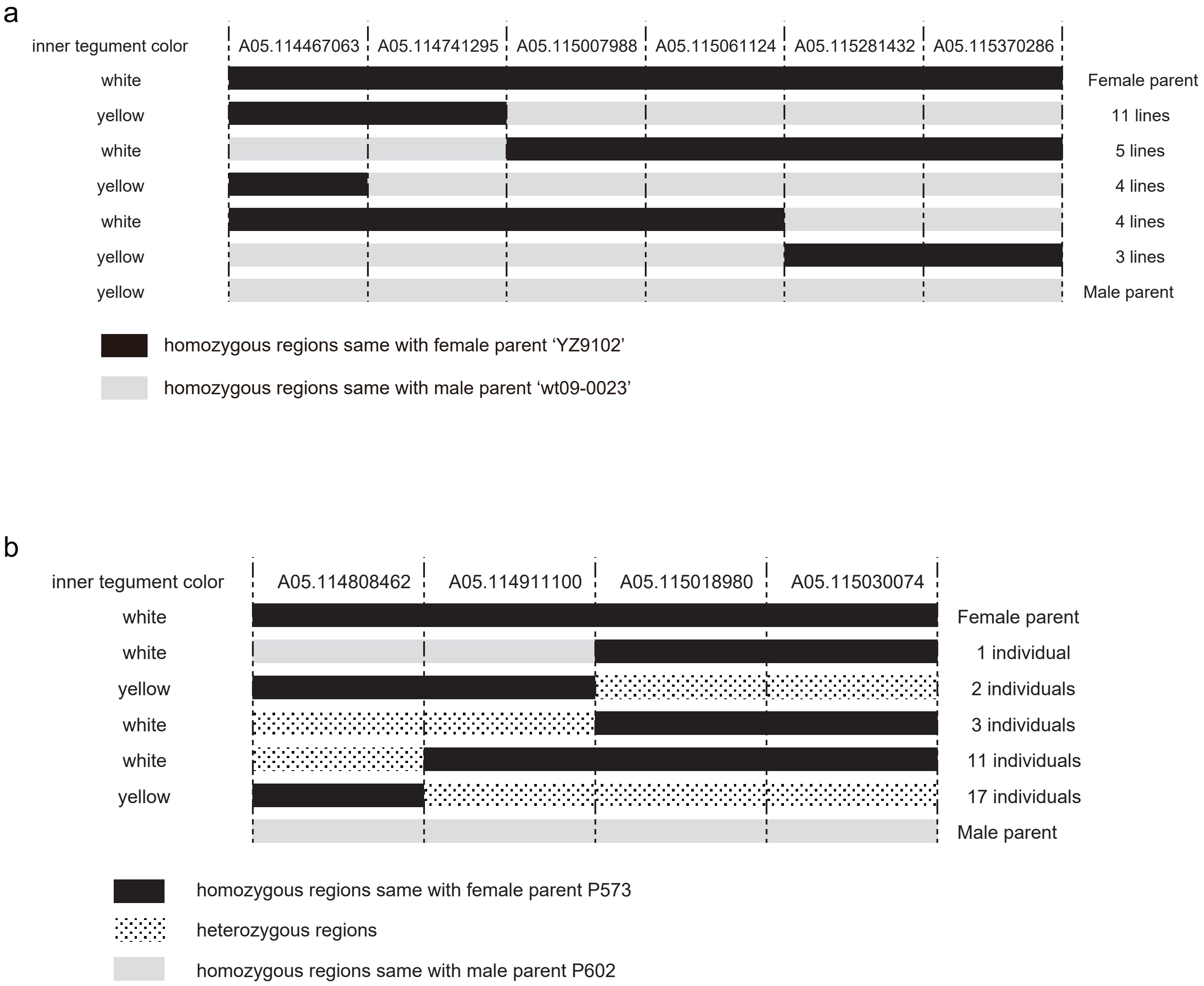

Supplementary Figure 10. Fine mapping of a QTL for the inner integument color (*qIICA05*). KASP markers used for fine mapping are named according to their chromosomal position on chr05. (a) Fine mapping based on from the YZ9102×wt09-0023 RIL population. Phenotypes and genotypes are reported for the parental lines and 27 recombinant RILs.
(b) Fine mapping based on the P573×P602 F2 population. Phenotypes and genotypes are reported for the parental lines and 34 recombinant individuals.

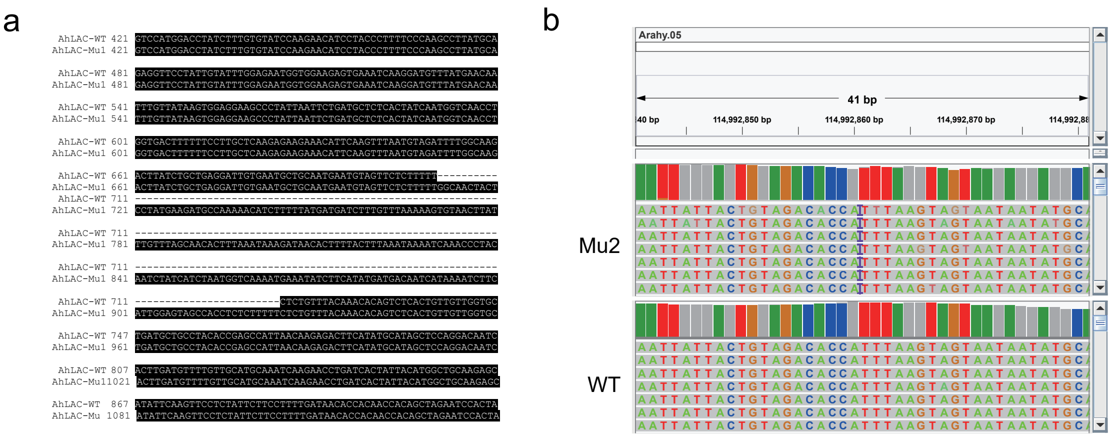

Supplementary Figure 11. Features of the mutations (Mu) identified for the gene AhLAC.
a) Sequence alignment showing the 214 bp insertion of the mutation type 1 (Mu 1); b) Integrative Genomics Viewer (IGV) image of the genomic region showing paired-end reads mapped on the candidate gene with a 1 bp insertion (purple I) described as mutation type 2 (Mu 2).

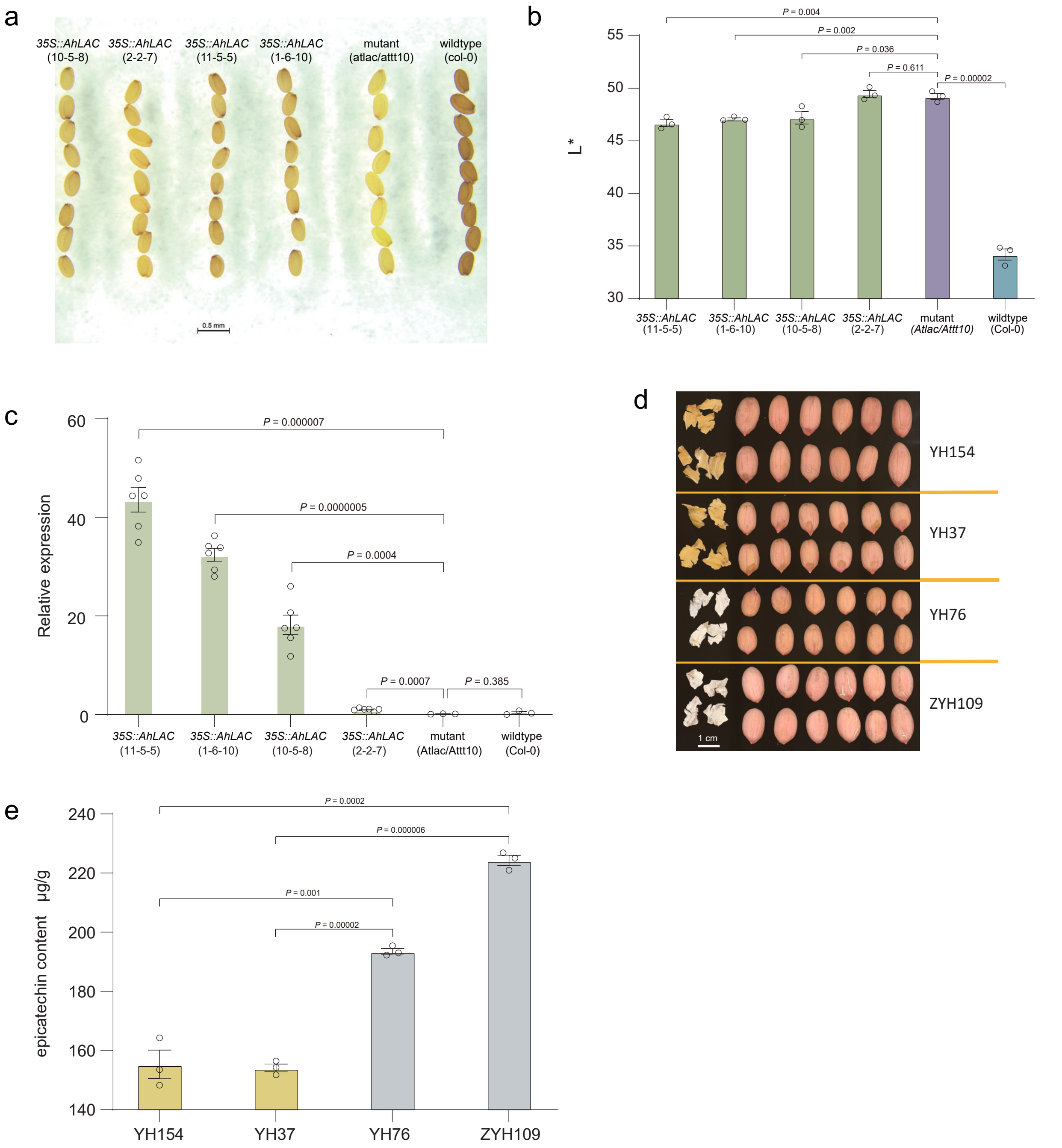

Supplementary Figure 12. *AhLAC* functional characterization. a-c) Seed phenotypes (a), lightness (L*) level (b) and *AhLAC* expression levels (c) in the *Arabidopsis* wild type line Col-0, the *atlac/att10* mutant line and four independent 35S::*AhLAC* transgenic lines in the mutant background; (d-e) Tegument color (d) and epicatechin content (e) of the peanut accessions YH154, YH37, YH76 and ZYH109. In b), c) and e), data are given as mean ± SEM; the two-tailed Student's *t* test was used to compare means. In the bar charts b) and e), n=3 biologically independent samples; In the bar chart c), n=6 biologically independent samples for the *35S::AhLAC* transgenic lines and n=3 biologically independent samples for the *Arabidopsis Atlac/Attt10* mutant and the wildtype.

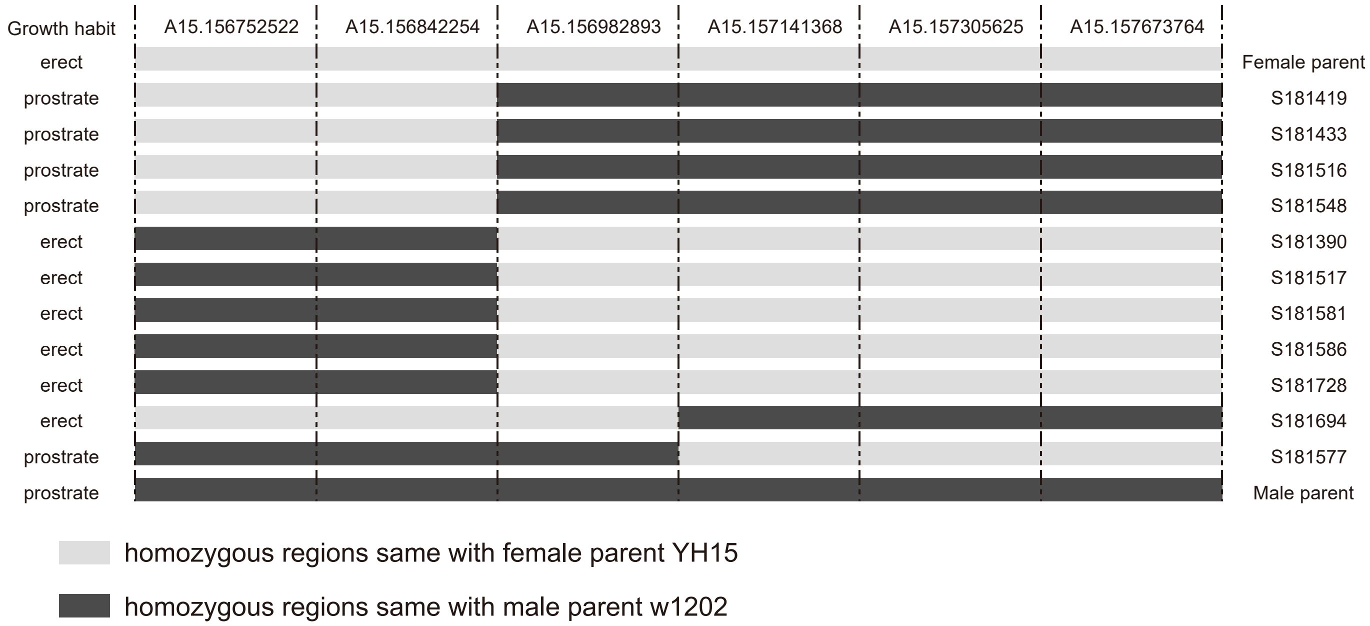

Supplementary Figure 13. Fine mapping of a QTL for the growth habit (*qGhA15*). KASP markers used for fine mapping are named according to their chromosomal position on chr15. Phenotypes and genotypes are reported for the parental lines and 11 recombinant RILs.

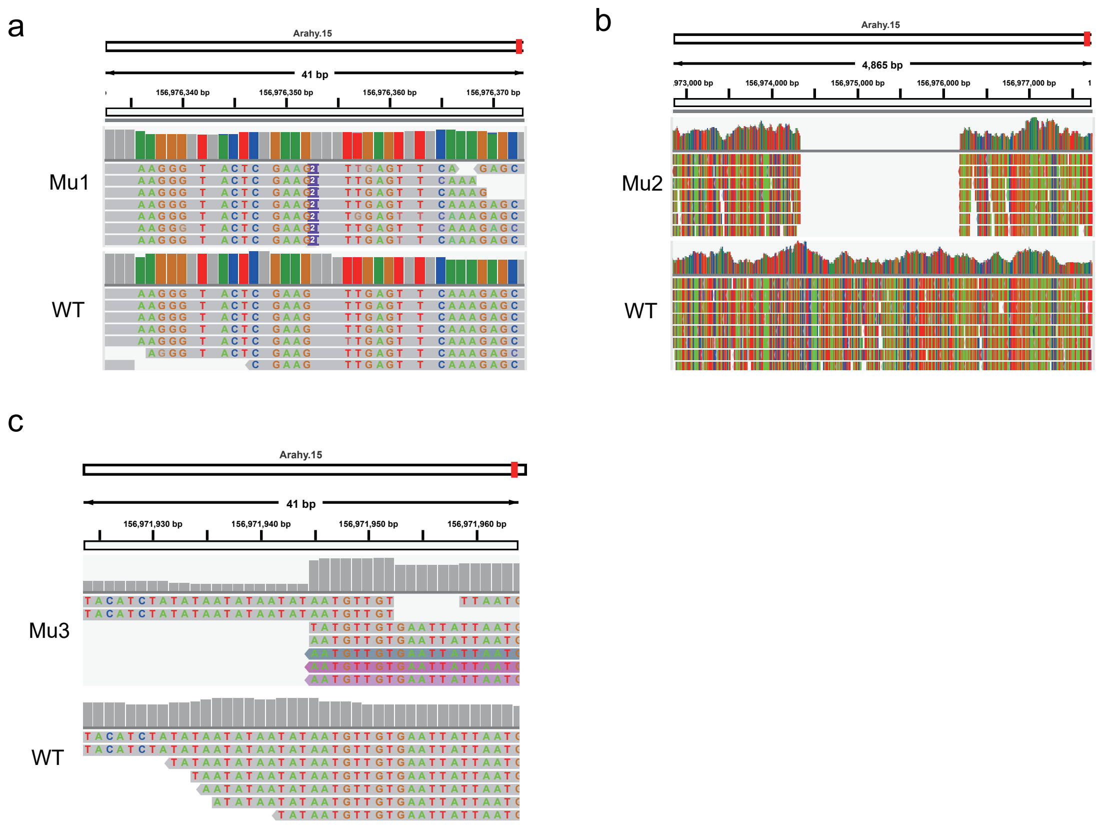

Supplementary Figure 14. Features of the mutations (Mu) identified for the gene *AhMADS-box transcription factor 6.* a) Integrative Genomics Viewer (IGV) image of the genomic region showing paired-end reads mapped on the candidate gene with a 2 bp insertion (purple 2) described as mutation type 1 (Mu 1); b) Integrative Genomics Viewer (IGV) image of the genomic region showing paired-end reads mapped on the candidate gene with a 1870 bp deletion described as mutation type 2 (Mu 2); c) Integrative Genomics Viewer (IGV) image of the genomic region showing paired-end reads mapped on the candidate gene with a MITE insertion described as mutation type 3 (Mu 3).

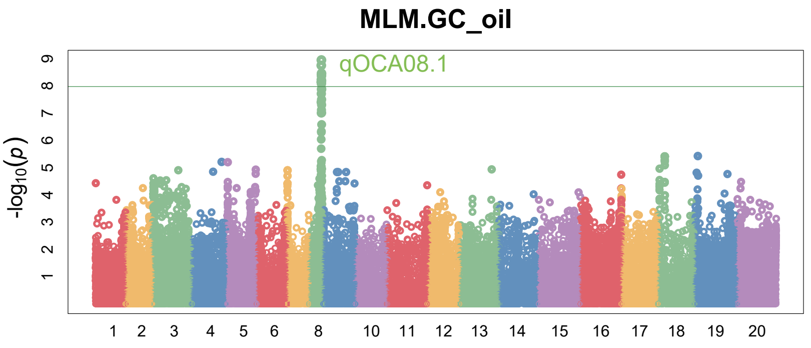

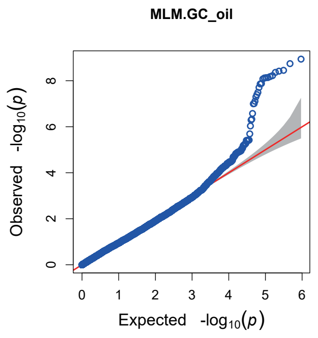

Supplementary Figure 15. GWAS for seed oil content. Manhattan plot (left) and quantile-quantile (Q-Q) plot (right). The mixed linear model (MLM) implemented in the R package GAPIT was used to test for marker trait association. The horizontal line in the Manhattan plot indicates the significance -log10(*P*) threshold for association after the Bonferroni correction. The shaded area in the Q-Q plot indicates the 95% confidence interval under the null hypothesis of no association between the SNPs and the trait, under the assumption of a uniform [0, 1] distribution for the P values.
